# Methicillin-resistant *Staphylococcus aureus* in Neonatal Intensive Care Unit

**DOI:** 10.3201/eid1103.040470

**Published:** 2005-03

**Authors:** Gili Regev-Yochay, Ethan Rubinstein, Asher Barzilai, Yehuda Carmeli, Jacob Kuint, Jerome Etienne, Mira Blech, Gill Smollen, Ayala Maayan-Metzger, Azita Leavitt, Galia Rahav, Nathan Keller

**Affiliations:** * Sheba Medical Center, Ramat-Gan, Israel; † Tel-Aviv University, Tel-Aviv, Israel; ‡ Tel-Aviv Medical Center, Tel-Aviv, Israel; § Faculté de Médecine Laennec, Lyon, France

**Keywords:** Community Methicillin-resistant *Staphylococcus aureus*, outbreak, neonatal intensive care unit, dispatch

## Abstract

A neonatal intensive care unit outbreak was caused by a strain of methicillin-resistant *Staphylococcus aureus* previously found in the community (ST45-MRSA-IV). Fifteen infected neonates were identified, 2 of whom died. This outbreak illustrates how a rare community pathogen can rapidly spread through nosocomial transmission.

Since 1998, strains of highly virulent, community-associated, methicillin-resistant *Staphylococcus aureus* (CA-MRSA), which are distinct from the typical nosocomial MRSA (NA-MRSA), have been reported (*1*,*2*). CA-MRSA is susceptible to numerous antimicrobial agents, in contrast to the multidrug-resistant (MDR) NA-MRSA phenotype, because it carries the staphylococcal cassette chromosome *mec* (SCC*mec*) type IV or V, rather than type I or III (*3*,*4*). The high virulence of CA-MRSA has been linked to Panton-Valentine leukocidin (PVL), a virulence factor found in most of these strains (*2*,*5*). We report a nosocomial MRSA outbreak in a neonatal intensive-care unit (NICU), by a non-MDR MRSA strain that carries the SCC*mec* type IV.

## The Outbreak

Sheba Medical Center is a 1,500-bed, tertiary care hospital in which ≈10,000 infants are delivered annually. MDR MRSA is endemic in the hospital, constituting 50%–60% of all *S. aureus* isolates, while non-MDR MRSA has been observed in only 2 cases in the last 5 years. The premature neonatal department admits 500 premature neonates annually, nearly 100% of whom are born in the hospital. The department contains ≈45 beds: 12–14 level 3 NICU beds in a single large space, 15 intermediate-intensive beds in a separate room, and 18 additional beds in 2 additional rooms. The NICU is separated by a corridor from the rest of the department. The nurse/patient ratio in the NICU is 1:3–1:5.

During October 2003, the microbiology laboratory identified MRSA blood isolates from 2 neonates in the NICU that were atypically susceptible to erythromycin, clindamycin, gentamicin, rifampicin (rifampin), trimethoprim-sulfamethoxazole (TMP-SMX), and ofloxacin (therefore designated non-MDR MRSA). Because of the rarity of such a phenotype in this institution, a retrospective search for all *S. aureus* clinical isolates from the premature neonatal department since 1998 was undertaken by using computerized laboratory data.

**Figure 1:**
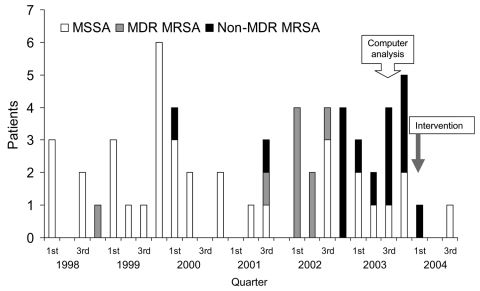
Incidence of *Staphylococcus aureus* infections in premature neonatal ward, 1998–2004.

A total of 14 neonatal infections with non-MDR MRSA were discovered during this period. A cluster of 12 cases was observed from October 2002 to December 2003 (designated the outbreak period), while 2 sporadic cases were isolated from January 1, 1998, to September 31, 2002, the preoutbreak period (Figure 1). The incidence of all *S. aureus* infections was 15.2 per 1,000 hospitalized neonates in the preoutbreak period, while during the outbreak period, it increased to 27.7 (p = 0.032). The incidence of non-MDR MRSA was 1.4 cases per 1,000 hospitalized neonates in the preoutbreak period; the rate increased to 18.5 cases per 1,000 hospitalized neonates (p < 0.0001) during the outbreak period (Table).

**Table:** Incidence rates of non–multidrug-resistant (MDR) methicillin-resistant *Staphylococcus aureus* (MRSA) and *S. aureus* infections: preoutbreak versus outbreak periods*

Infection	No. infected patients per 1,000 hospitalized patients	p value
Non-MDR MRSA, preoutbreak period	1.4	<0.0001
Non-MDR MRSA, outbreak period	18.5	
All *S. aureus*, preoutbreak period	15.2	0.031
All *S. aureus*, outbreak period	27.7	
Other *S. aureus* (methicillin-susceptible and MDR MRSA), preoutbreak period	13.8	0.38
Other *S. aureus* (methicillin-susceptible and MDR MRSA), outbreak period	9.2	

*Preoutbreak period, January 2000–September 2002; outbreak period, October 2002–December 2003.

**Figure 2:**
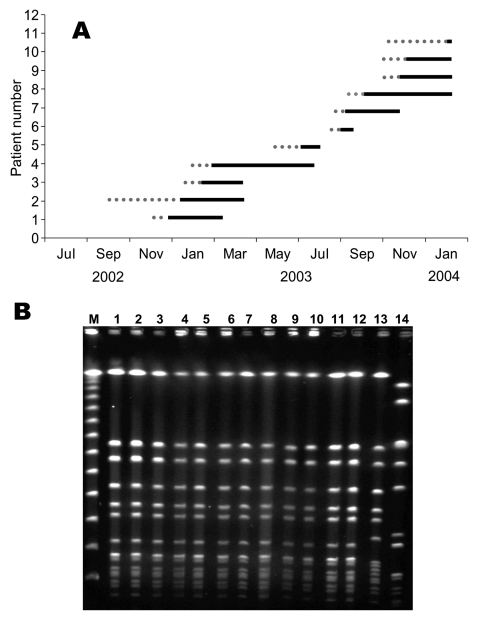
A) Nosocomial transmission of non–multidrug-resistant (MDR) methicillin-resistant *Staphylococcus aureus* (MRSA) in the premature neonatal ward. Dotted lines represent hospitalization until first non-MDR MRSA isolation. Solid lines represent hospitalization after first non-MDR MRSA isolation until discharge. B) pulsed-field gel electrophoresis of MRSA outbreak isolates; lanes 1–10: ST45-MRSA-IV outbreak strains isolated from neonates; lanes 11–12: ST45-MRSA-IV outbreak strains isolated from nurses; lane 13: ST45 methicillin-susceptible *S. aureus* strain from a community survey; lane 14: common nosocomial MRSA strain ST5-MRSA-II.

The first case of the cluster occurred 15 months after the second sporadic case. This case was in a 690-g female neonate of 25 weeks’ gestational age, hospitalized in the NICU since birth. A non-MDR MRSA was isolated from eye discharge on her 22nd day of life, and she recovered with no antimicrobial drug treatment. The next 11 case-patients (Figure 2A) were also hospitalized in the NICU since birth; 9 were bacteremic and had signs of sepsis, 4 had sputum isolates (2 of these patients had pneumonia), 2 died, and 1 death was attributable to the infection. Thirteen of 14 neonates were treated with vancomycin for periods ranging from 6 to 48 days; 2 were treated with vancomycin and rifampicin. None of the patients had perinatal infections, and the median age when infection was diagnosed was 30 days (range 11–115 days). Eleven patients had extremely low birth weight (range 568–2,440 g, median 810 g), and 12 had low gestational age (range 23–35 weeks, median 25 weeks). All patients had indwelling devices (mechanical ventilation for 11 to 74 days, central lines and total parenteral nutrition for 7–38 days). Four neonates had previous major surgery, and all neonates had at least 1 of several complications of prematurity (respiratory distress syndrome, bronchopulmonary dysplasia, intraventricular hemorrhage, retinopathy of prematurity).

An infection control intervention was initiated on December 5, 2003. Screening for *S. aureus* carriage was performed by nasal and umbilical sampling of all hospitalized neonates (N = 30; all were sampled on day 1) and nasal sampling of all the department healthcare workers (N = 114 [47 nurses, 30 physicians, 37 other healthcare workers]; 85% were sampled within 5 days). Swabs were streaked onto a differential media (CHROMagar plates, HyLabs, Rehovot, Israel) and incubated for 24 to 48 hours at 35°C. Suspicious colonies were then conventionally identified. Oxacillin resistance was determined according to National Committee for Clinical Laboratory Standards recommendations (*6*) and was verified by polymerase chain reaction (PCR) for the presence of *mecA* (*1*). Eight patient-unique, non-MDR *S. aureus* blood isolates from the previous year were also available for analyses.

In this point-prevalence surveillance, a non-MDR MRSA was isolated from 3 neonates with previous clinical isolates and from 4 newly recognized carriers. Thus, 7 (23.3%) of 30 hospitalized neonates were carriers. In addition, 2 (1.7%) healthcare workers, both nurses (4.3% of nurses), carried this strain; MDR MRSA was not isolated. Methicillin-susceptible *S. aureus* was carried by 28.1% of healthcare workers and was not isolated from any of the neonates.

Pulsed-field gel electrophoresis (PFGE) was performed, as previously described (*7*), on 13 unique isolates from 11 neonates and 2 nurses. A single identical outbreak strain was identified by the PFGE pattern (Figure 2B). All non-MDR MRSA were found to be SCC*mec* type IV by a modification of the method described by Oliveira (*8*). By using multilocus sequence typing (MLST) (*9*), the outbreak strain was classified as ST45-MRSA-IV. PCR screening of the outbreak strain for 25 virulence factor genes was performed as described by Jarraud et al. (*10*). MLST and PCR of virulence factors were performed on 8 outbreak isolates. Virulence factors commonly found in CA-MRSA, *lukS*-PV-*lukF*-PV encoding PVL components S and F, γ-hemolysin variant (*hlg-v*), and *agr* type 3, were not detected in the outbreak strain. The outbreak strain was positive for enterotoxin gene cluster (*egc*), γ-hemolysin (*hlg*), and *agr* type 1 and had similar PFGE and MLST results as the major methicillin-susceptible *S. aureus* (MSSA) strain found in the community served by this hospital. The outbreak strain was identical to the CA-MRSA that is rarely isolated in healthy carriers in Israel (*11*). Moreover, this strain differed from the MDR MRSA strains that are commonly isolated in this medical center (see Figure 2B).

After initial surveillance, all case-patients were isolated and cohorted, appropriate hand hygiene practices were reinforced for healthcare workers, and all neonates were bathed with diluted (1:10) chlorhexidine gluconate 4% once daily for 3 consecutive days. Nasal mupirocin was implemented 3 times per day for 5 consecutive days for all carriers. These regimens were well tolerated with no adverse events.

Three weeks after the first surveillance and intervention, a second surveillance was conducted in the NICU and intermediate neonatal care unit. No new cases were identified. Of the 7 carriers, only 4 were still hospitalized. Of these, 2 continued to carry the outbreak strain, and 2 were successfully decolonized. The 2 carriers were decolonized after a second course of a similar regimen (chlorhexidine bath followed by mupirocin administration).

The 2 colonized nurses were sampled twice, 1 week apart, and were found to be persistent carriers of the outbreak strain. They were instructed about good hand hygiene practices, and nasal mupirocin was recommended for 5 days. One nurse cleared her nasal non-MDR MRSA after mupirocin treatment and acquired a new strain of MSSA 2 weeks later. The other refused mupirocin treatment and persistently carried the outbreak strain for 8 weeks. During this period, despite our instructions, she continued to work until she went on maternity leave. Before she returned to work 12 weeks later, she was decolonized by using nasal mupirocin. In a 7-month follow-up, no new cases of non-MDR MRSA were identified (Figure 1).

## Conclusions

This report documents a nosocomial outbreak of a non-MDR, PVL-negative MRSA strain, ST45-IV, in a NICU. This strain is clearly distinct from the NA-MRSA strains in this medical center, but it is identical to a CA-MRSA strain previously isolated in the community (in 0.5% of *S. aureus* carriers) and is nearly identical to the major MSSA strain circulating in this community (*11*). We hypothesize that the outbreak strain evolved in the community and penetrated into the NICU. Two previous reports of possible CA-MRSA in NICUs have been reported; however, neither characterized the alleged pathogen genetically (*12*,*13*).

The outbreak strain reported here is similar to CA-MRSA strains described in Europe, the United States, and Australia in that it is susceptible to many antimicrobial drugs and carries the SCC*mec* type IV (*2*,*5*). However, this strain is distinct from those strains because it lacks the PVL components S and F, as well as other virulence factors. Indeed, skin and soft-tissue infections typically described as caused by CA-MRSA (*1*,*2*) were not observed in any of the neonates in this outbreak.

MRSA outbreaks in NICUs have been reported to be difficult to contain (*12*,*14*). Only implementation of aggressive infection control measures, frequently combined with mupirocin treatment, has been successful in controlling such outbreaks. The outbreak described here was similarly contained by implementing a multifaceted infection control intervention. Since all the measures were undertaken simultaneously, we cannot define which of the measures was the most important.

We assume that the primary source of this outbreak was either a parent of the index patient or a carrier nurse. Because of a low ratio of nurses to neonates in the NICU and the high contact rate each nurse had with neonates in that facility, we could not trace specific contact patterns or perform a case-control study to further investigate this outbreak.

The difficulties encountered in implementing proper infection control measures are demonstrated by the fact that 1 healthcare worker continued to be engaged in patient care for 8 weeks, despite continued colonization by the outbreak strain, against our advice to her supervisors. Fortunately, this action did not result in persistence of the outbreak.

This outbreak illustrates the penetration of a community pathogen into the hospital, where nosocomial transmission, particularly in an intensive care setting, may rapidly spread the pathogen. The susceptibility of newborns, coupled with insufficient infection control measures and inadequate nurse-to-patient ratio, contributed to this outbreak. We call for specific attention to the possibility of “reverse penetration” of community MRSA strains becoming nosocomial pathogens.
